# Use of multikinase inhibitors/lenvatinib in patients with high cardiovascular risk/vasculopathy and radioiodine refractory‐differentiated thyroid cancer

**DOI:** 10.1002/cam4.5127

**Published:** 2022-10-06

**Authors:** Paula Jimenez‐Fonseca

**Affiliations:** ^1^ Department of Medical Oncology, Hospital Universitario Central de Asturias Instituto de Investigación Sanitaria del Principado de Asturias (ISPA) Oviedo Spain

**Keywords:** cardiovascular disease, hypertension, lenvatinib, multikinase inhibitors, sorafenib, thyroid carcinoma

## Abstract

Antiangiogenic tyrosine kinase inhibitors are the treatment of choice in radioiodine refractory‐differentiated thyroid cancer (RR‐DTC). Nevertheless, these therapies present class toxicities that may impact their feasibility and patient's quality of life. Their mechanism of action explains the high prevalence of hypertension associated with their use, which reaches 68% with lenvatinib. Moreover, up to 85% of patients treated in the SELECT clinical trial were receiving baseline antihypertensive treatment. These data support the need for prevention, detection, and early management of hypertension. Prevention can be accomplished by controlling cardiovascular risk factors (hypertension, diabetes, obesity, and dyslipidemia) and those associated with lifestyle (smoking, harmful alcohol consumption, and physical inactivity) and electrolyte disorders. It is necessary to achieve stabilization of cardiovascular diseases. Detection involves baseline measurement and monitoring of blood pressure and cardiac function. Treatment requires optimization of baseline blood pressure and early initiation of antihypertensive agents.

## Graphical abstract

Antiangiogenic tyrosine kinase inhibitors, the treatment of choice for radioiodine‐refractory differentiated thyroid cancer (RR‐DTC), are associated with a high prevalence of hypertension. Therefore, there is a need for prevention, detection, and early management of hypertension in these patients. Treatment requires optimization of baseline blood pressure and early initiation of antihypertensive agents.

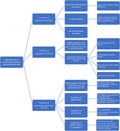

## 1 INTRODUCTION

Differentiated thyroid cancer (DTC) is the most common endocrine neoplasm, and improvement in diagnostic imaging has contributed to the increase in incidence.^1^, ^2^ Simultaneously, there has been an increase in the incidence of advanced cancers (>5% of all DTCs), highlighting the need to identify markers of indolent versus aggressive disease to improve the ability to tailor treatment strategies based on an individual's thyroid cancer biology.^2^

While most patients with advanced disease can be treated with radioiodine, a variable percentage, depending on the follow‐up period, will become refractory and eventually require systemic antineoplastic treatment. The prognosis worsens in these patients with 10‐year survival rates decreasing from 95% for most patients diagnosed with DTC to 10% for those with no evidence of iodine uptake.^3^, ^4^ To date, the multi‐targeted kinase inhibitors (MKIs) targeting the vascular endothelial growth factor (VEGF) signaling pathway, sorafenib (randomized controlled trial [RCT] DECISION), lenvatinib (RCT SELECT), and cabozantinib (RCT COSMIC‐311) are the cornerstone of treatment for advanced radioactive iodine (RAI) refractory‐DTC (RR‐DTC).^5^, ^6^, ^7^ Table 1 summarizes the characteristics and results of the three RCTs. These MKIs have demonstrated an increase in objective response rate (ORR) and progression‐free survival (PFS) without a confirmed overall survival benefit that may have been influenced by crossover allowed in their registration clinical trials. This has led to the recommendation of treatment with MKIs in patients with unresectable advanced RR‐DTC who have a significant tumor burden or progressive cancer, especially in the presence of symptoms.^3^, ^8^ In contrast, the latest American Thyroid Association guidelines suggest delaying the initiation of systemic treatment in patients with stable or minimally progressive disease given the risk of serious adverse effects with MKIs.^8^

**TABLE 1:** Characteristics and results of phase 3 randomized clinical trials DECISION, SELECT, and COSMIC‐311 in advanced radioactive iodine refractory‐differentiated thyroid cancer

Phase 3 RCT	DECISION		SELECT		COSMIC‐311	
Drug	Sorafenib	Placebo	Lenvatinib	Placebo	Cabozantinib	Placebo
Population	*N* = 207	*N* = 210	*N* = 261	*N* = 131	*N* = 125	*N* = 62
International	North America, Europe, and Asia		America, Europe, Asia, and Australia		North America, Europe, Asia, and others	
Recruitment period	2009–2011		2011–2012		2019–2020	
Type of tumor	RR‐DTC		RR‐DTC		RR‐DTC	
Radiologic evidence of progression	Within 14 months		Within 13 months		During or following VEGFR‐targeted therapy	
Progressed after	One RAI therapy within 16 months		One RAI therapy within 12 months		Sorafenib or/and lenvatinib	
Line of systemic treatment	First line		First and second line		Second and third line	
FDA approval	2013		2015		2021	
PFS, months^a^	10.8^a^	5.8	18.3^a^	3.6	Not reached	1.9
HR (CI)	0.59 (95% CI, 0.45–0.76)		0.21 (99% CI, 0.14–0.31)		0.22 (96% CI, 0.13–0.36)	
*p*‐value	<0.0001		<0.001		<0.0001	
Response rate	12.2%	0.9%	64.8%	1.5%	15%^a^	0%
OR (95% CI)	‐		28.87 (12.46–66.86)		‐	
*p*‐value	<0.0001		<0.001		=0.028	
OS	Not reached	Not reached	Not reached	Not reached	Not reached	Not reached
HR (95% CI)	0.80 (0.54–1.19)		0.73 (0.5–1.07)		0.54 (0.27–1.11)	
*p*‐value	= 0.14		= 0.10			
Hypertension						
Any grade	40.6%		67.8%	9.2%	19% (G1‐2)	2% (G1‐2)
Grade ≥3	9.7%		41.8%	2.3%	8% (G3) 1% (G4)	3% (G3)
Proteinuria	Not reported					
Any grade			31%	1.5%	14% (G1‐2)	3% (G1‐2)
Grade ≥3			10%	0%	1% (G3)	0% (G3)
QT/QTc interval prolongation	Not reported				Not reported	
Any grade			8.8%			
Grade ≥3			1.5%			
Heart failure	Not reported				**Cardiac arrest** 1% (G5)	**Cardiac arrest** 2% (G5)
Any grade			6.5%			
Grade ≥3			1.5%			
VTE	Not reported				**PE** 2% (G1‐2) 2% (G3) 1% (G5) **DVT** 2% (G1‐2) 1% (G3)	**PE** 0% (G1‐2) 0% (G3‐5) **DVT** 0% (G1‐2) 0% (G3‐5)
Any grade			5.4%			
Grade ≥3			3.8%			
Dose intensity	81% (651 mg/d)		72% (17.2 mg/d)		70% (42 mg/d)	
Dose interruptions	66.2%		82.4%		‐	
Dose reductions	64.3%		67.8%		56%	
Treatment discontinuations	18.8%		14%		5%	

Abbreviations: CI, confidence interval; DVT, deep vein thrombosis; FDA, Food and Drug Administration; G, grade of toxicity; HR, hazard ratio; PE, pulmonary embolism; PFS, progression‐free survival; RCT, randomized clinical trial; RR‐DCT, radioactive iodine refractory‐differentiated thyroid cancer; OR, odds ratio; OS, overall survival; VEGFR, vascular endothelial growth factor receptor; VTE, venous thromboembolic event

^a^ Primary endpoint.

VEGF blockade induces vasoconstriction, which explains why the most common class adverse effect is hypertension, present in 68% of patients taking lenvatinib and 41% taking sorafenib in the SELECT and DECISION phase III trials, respectively.^5^, ^6^, ^9^ Hypertension and other cardiovascular adverse events (AEs) are the main disadvantages of antiangiogenic therapy. The problem of anti‐VEGF therapy overlaps with the rise of cardiovascular disease (CVD) and obesity in Western populations in recent years. CVD remains the leading cause of morbidity and mortality worldwide and is associated with the presence of cardiovascular (CV) risk factors in many adults.^10^ The higher the level of each CV risk factor and the more risk factors an individual has, the greater the likelihood of developing CVD, with hypertension being the major risk factor.^10^, ^11^

In a post hoc study, hypertension was significantly correlated with improved efficacy of lenvatinib in this population.^12^ In turn, hypertension caused by MKIs may exacerbate CV risk factors and CVD associated with many thyroid cancer patients.

## 2 HISTORICAL PERSPECTIVE

In RR‐DTC, sorafenib and lenvatinib are indicated for the first‐line treatment of advanced unresectable disease,^5^, ^6^ whereas cabozantinib is indicated in patients who have received previous lenvatinib or sorafenib and progressed during or after treatment with up to two VEGFR tyrosine kinase inhibitors.^7^ Sorafenib was the first MKI approved by the Food and Drug Administration (FDA) for the treatment of patients with locally recurrent or metastatic, progressive, RR‐DTC in 2013. The DECISION RCT showed a PFS with sorafenib vs placebo of 10.8 vs. 5.8 months (hazard ratio [HR], 0.59; 95% confidence interval [CI] 0.45–0.76; *p* < 0.0001) and a 12% response rate with sorafenib.^6^ Lenvatinib confirmed an ORR of 65% and PFS of about 18 months compared to 3.6 months with placebo (HR, 0.21; 99% CI, 0.14–0.31; *p* < 0.001) in the SELECT RCT.^5^ These data allowed its approval by the FDA for the treatment of patients with metastatic RR‐DTC in 2015.^13^

In the systemic treatment of RR‐DTC, the toxicity of these MKIs can compromise drug continuity and dose intensity and thus affect therapeutic outcomes.^14^ Patients treated with lenvatinib in the SELECT RCT maintained treatment for 75% (13.8 months) of the time to progression (PFS, 18.3 months) and dose intensity was 72% (17.2 mg/day) of the standard dose leading to two to three levels of reduction. Dose interruptions, dose reductions, and treatment discontinuations were needed in 82.4%, 67.8%, and 14% of the patients, respectively.^5^ This highlights the importance of preventing and early management of toxicity to try to maintain treatment at full doses for the longest possible duration.

AEs related to CVD with lenvatinib were hypertension (67.8%; grade ≥3, 41.8%), proteinuria (31%; grade ≥3, 10%), arterial or venous thromboembolic events (5.4%; grade ≥3, 2.7%; stroke [1.1%], myocardial infarction [0.9%], and transient ischemic attack [0.7%]), or venous thromboembolic events (5.4%; grade ≥3, 3.8%), QT/QTc interval prolongation (8.8%; grade ≥3, 1.5%), and heart failure (6.5%; grade ≥3, 1.5%).^5^ The toxicities that most frequently led to discontinuation of lenvatinib were asthenia, hypertension (1.1%), proteinuria, stroke, diarrhea, and pulmonary embolism, and those that caused dose interruption or reduction were diarrhea and hypertension.

The incidence of hypertension with sorafenib in the DECISION RCT was lower than that reported in the lenvatinib study (40.6% of any grade; 9.7% grade ≥3).^5^, ^6^ For early detection and management, it is important to know that hypertension and proteinuria occur early in lenvatinib treatment, with a median of 16 and 6.7 days to onset and most grade 3–4 AEs occur during the first 6 months of treatment.^12^, ^15^

The only CV AE reported in the phase 3 RCT with sorafenib, lenvatinib, and cabozantinib in other malignancies (advanced hepatocarcinoma and renal cell carcinoma) was hypertension (Table 2).^16^, ^17^, ^18^ Hypertension of any grade, and severe with sorafenib, was similar in frequency in the DECISION (DTC) and REFLECT (hepatocarcinoma) RCTs and lower in the SHARP (hepatocarcinoma) RCT.^6^, ^16^, ^17^ The starting dose was the same in all three studies and the dose intensity was similar. The SHARP RCT with sorafenib in advanced hepatocarcinoma found that 3% of patients had cardiac ischemia or infarction.^16^ The lenvatinib group in the REFLECT RCT had lower hypertension and proteinuria rates than in the SELECT RCT, which is explained by the starting doses of 8–12 mg and 24 mg, respectively.^5^, ^17^ In previously treated patients, cabozantinib resulted in similar rates of hypertension, dose intensity, dose reduction, and discontinuation in the advanced renal cancer RCT (METEOR) as in the DTC RCT (COSMIC‐311).^7^, ^18^

**TABLE 2:** Cardiovascular effects of sorafenib, lenvatinib, and cabozantinib in phase 3 randomized clinical trials in advanced differentiated thyroid cancer (DECISION, SELECT, and COSMIC‐311), hepatocarcinoma (SHARP and REFLECT), and renal carcinoma (METEOR)

Phase 3 RCT	DECISION	SHARP	REFLECT	SELECT	REFLECT	COSMIC‐311	METEOR
Drug	Sorafenib	Sorafenib	Sorafenib	Lenvatinib	Lenvatinib	Cabozantinib	Cabozantinib
Dose	800 mg/d	800 mg/d	800 mg/d	24 mg/d	≥60 kg: 12 mg/d; <60 kg: 8 mg/d	60 mg/d	60 mg/d
Population	*N* = 207	*N* = 299	*N* = 476	*N* = 261	*N* = 478	*N* = 125	*N* = 330
Type of tumor	RR‐DTC	Advanced hepatocellular carcinoma	Advanced hepatocellular carcinoma	RR‐DTC	Advanced hepatocellular carcinoma	RR‐DTC	Advanced renal cell carcinoma
Line of systemic treatment	1st line	1st line	1st line	1st‐2nd line	1st line	2nd‐3rd line	≥2nd line
Hypertension							
Any grade	40.6%	5%	30.3%	67.8%	42.2%	19% (G1‐2)	37%
Grade ≥3	9.7%	2%	14.3%	41.8%	23.3%	8% (G3) 1% (G4)	15%
Proteinuria	Not reported	Not reported					Not reported
Any grade			24.6%	31%	11.4%	14% (G1‐2)	
Grade ≥3			5.7%	10%	1.7%	1% (G3)	
Heart failure	Not reported		Not reported				Not reported
Any grade		Cardiac ischemia or infarction		6.5%	0.6%	Cardiac arrest	
Grade ≥3		All grades: 3%		1.5%	0.4%	1% (G5)	
QT prolongation							
Any grade				8.8%	6.9%		
Grade ≥3				1.5%	2.4%		
Dose intensity	81% (651 mg/day)	80%	83% (663.8 mg/d)	72% (17.2 mg/d)	87% (10.5 mg/d)	70% (42 mg/d)	73% (44 mg/d)
Dose interruptions	66.2%	44%	32.2%	82.4%	39.9%	‐	
Dose reductions	64.3%	26%	38.1%	67.8%	37%	56%	60%
Treatment discontinuations	18.8%	38%	7.2%	14%	8.8%	5%	9%

Abbreviations: G, grade of toxicity; RCT, randomized clinical trial; RR‐DCT, radioactive iodine refractory‐differentiated thyroid cancer.

## 3 CURRENT SITUATION

Before initiating MKIs, it is essential to evaluate the suitability of patients who are candidates to receive them, as well as their performance status, functional reserve, and comorbidities.^11^ Within this evaluation, it is necessary to identify patients at increased risk of CV toxicity through a careful assessment of CV risk factors and previous CVD to detect subclinical cardiac abnormalities.^19^, ^20^, ^21^ Risk assessment should include physical examination, family history of premature CVD (younger than 50 years), CV risk factors such as hypertension, diabetes, obesity, dyslipidemia, and those associated with lifestyles such as smoking, harmful alcohol consumption, and physical inactivity.^22^

A baseline measurement of cardiac function by electrocardiogram, imaging studies (echocardiography, ejection fraction), and cardiac biomarkers (natriuretic peptides or troponins) should be performed to allow adequate interpretation of changes during the follow‐up.^20^ These diagnostic tests should be repeated every 6 months and whenever the patient has any symptoms, especially in patients with known CVD. The baseline blood pressure (BP) should be <140/90 mmHg and frequent BP monitoring should be performed throughout treatment in all patients. Regular urinalysis should be conducted to detect the onset of proteinuria.

Patients with concomitant diseases (e.g., CVD, renal, or hepatic insufficiency), prolonged QT interval, high risk of bleeding, with a body weight <60 kg, or those with poor performance status have lower tolerability to lenvatinib and MKIs, in general, as well as a higher risk of CV events or complications from hypertension. Elderly age, Asian race, obesity, high sodium intake, alcohol abuse, smoking, or reduced physical activity are also risk factors for MKI‐induced hypertension.^23^, ^24^ Certain genetic polymorphisms in VEGF or markers (EGLN3, EGF, and WNK1) have been associated with an increased risk of hypertension in patients treated with other antiangiogenic agents such as bevacizumab or sunitinib.^24^ Therefore, these patients at high risk of developing cardiotoxicity should be examined by a cardiologist with expertise in this field or by a specialist in cardiooncology and should be closely monitored to prevent and detect possible toxicity or worsening of their disease.^25^

The importance of early management of hypertension before starting MKIs is due to its high prevalence and its association with CVD. In fact, 56% of patients who participated in the SELECT RCT were receiving antihypertensive treatment before starting the study drug, and 68% were receiving antihypertensive therapy concomitant with lenvatinib at the end of the study. An analysis of AEs indicated a potential association between treatment‐emergent hypertension and an increased likelihood of developing congestive heart failure (CHF). However, the development of CHF did not appear to be influenced by either the duration of lenvatinib treatment or the severity of treatment‐emergent hypertension.^26^ Similarly, in patients with baseline CVD, hypertension usually takes less time to affect the patient's well‐being or induce life‐threatening conditions.^14^, ^19^, ^26^

## 4 TREATMENT RECOMMENDATIONS

**FIGURE 1:**
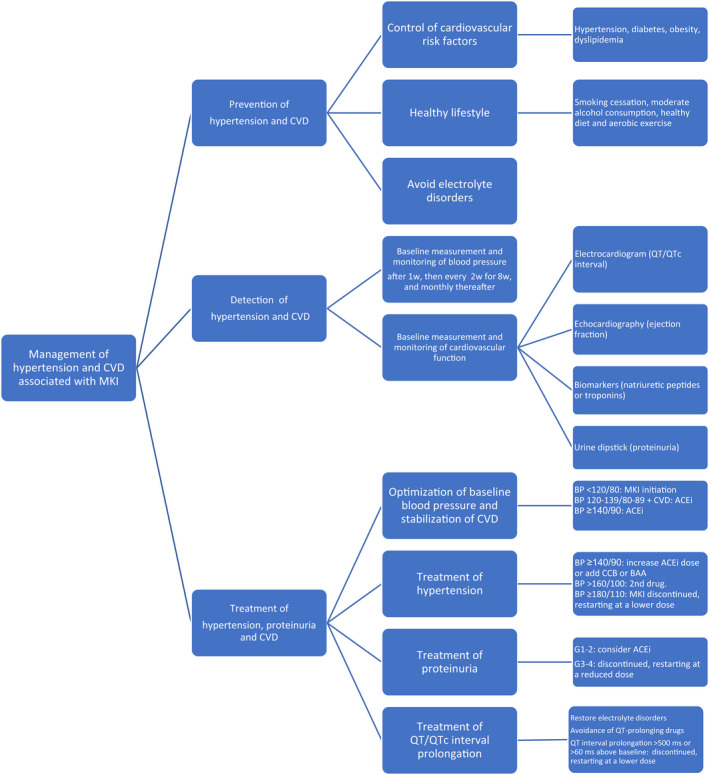
Management of hypertension, proteinuria, and CVD associated with MKI‐targeting VEGF. ACEi, angiotensin‐converting enzyme inhibitor; BAA, beta‐adrenoceptor antagonist; BP, blood pressure; CCB, calcium channel blocker; CVD, cardiovascular disease; G, grade of toxicity; MKI, multikinase inhibitor; w, week.

Since CV toxicity and specifically hypertension is the most frequent toxicity with lenvatinib and sorafenib, prevention, detection, and intensive management is important (Figure 1).^5^, ^6^

Lenvatinib is indicated at an initial dose of 24 mg/day in patients with controlled CVD and, in the case of an intolerable or refractory AE, stepwise dose reductions are recommended, as indicated in its Summary of Product Characteristics.^24^ A washout period of about 4 weeks is recommended if the patient received sorafenib or other prior systemic antineoplastic therapy.^5^, ^26^ Lenvatinib has not been studied in patients who have had an arterial thromboembolism or CV event in the previous 6 months or have unstable hypertension and should therefore be used with caution in such patients.^5^, ^26^

In all patients, a lifestyle that includes a healthy diet, smoking cessation, moderation of alcohol consumption, regular aerobic exercise, and weight control should be promoted.^14^ When the baseline risk of cardiotoxicity is high due to preexisting CVD, previous MKI, or CV risk factors, risk factor control should be optimized and prophylaxis with cardio‐ and reno‐protective drugs (angiotensin‐converting enzyme inhibitor [ACEi], angiotensin II receptor blocker [ARB], beta‐adrenoceptor antagonists [BAA], and statin) should be considered.^19^, ^25^, ^27^

BP monitoring under MKI treatment should be performed after 1 week, then every 2 weeks for the first 8 weeks, and monthly thereafter. Hypertensive patients should be taking a stable dose of an antihypertensive drug for at least 1 week before initiating lenvatinib.^28^ If baseline BP is <120/80 mmHg, MKIs should be initiated. If prehypertension values are systolic BP 120–139 mmHg or/and diastolic BP 80–89 mmHg, MKI should be initiated in patients without CVD or CV risk factors, and in those with a CV history, antihypertensive drugs should be started 1 week before initiation of MKI. If at baseline or during treatment, BP is ≥140/90 mmHg, an ACEi or ARB should be started and, in patients already taking antihypertensive drugs, the dose can be increased, as appropriate.^27^ When monotherapy is insufficient to reduce BP, a different class of antihypertensive such as a calcium channel blocker (CCB), or BAA can be added; the latter is preferred in patients with ischemic heart disease.^19^ BP should be evaluated at 2 weeks.

If BP is >160/100 mmHg despite optimization of antihypertensive therapy, a second drug is recommended and, if it does not reduce BP, lenvatinib should be interrupted until BP is reduced and resumed at a reduced dose. If BP remains elevated after dose reduction, a third antihypertensive drug such as long‐acting nitrate should be prescribed. If hypertension becomes severe (≥180/110 mmHg), lenvatinib should be discontinued.

The presence of proteinuria should be examined at baseline and periodically during treatment by urine dipstick and, when found to be 2+ or more, 24‐hour urine collection should be performed to measure urinary protein and/or urinary protein/creatinine ratio. In patients with renal insufficiency, especially those caused by CV risk factors such as hypertension, proteinuria should be carefully monitored and treated. When a patient presents with grade 1–2 proteinuria, lenvatinib should be maintained.^27^ In addition, ACEi or ARB can be administered; the second choice would be a CCB such as amlodipine, nitrates, or BAA while nondihydropyridine drugs such as verapamil or diltiazem should be avoided due to potential interaction via cytochrome P450 3A4 metabolism. When proteinuria is grade 3–4 (urinary protein ≥3.5 g/day or urinary protein/creatinine ratio ≥3.5), or if grade 1–2 occurs in patients at high risk for CV, renal pathology, associated with edema, or elevated serum creatinine, lenvatinib should be discontinued. Once proteinuria has been reduced below 2 g/day, lenvatinib should be restarted at a reduced dose.^27^

Electrolyte disorders (e.g., hypokalemia, hypocalcemia, hypomagnesemia) that increase the risk of QT/QTc interval prolongation should be monitored and corrected prior to initiating lenvatinib. Electrolytes (magnesium, potassium, and calcium) should be monitored monthly during treatment and loss should be restored as needed. Concomitant administration of lenvatinib with drugs that could increase QT interval prolongation (e.g., ivabradine, mifepristone, probucol, vinflunine) is contraindicated. Lenvatinib should be discontinued in case of QT interval prolongation >500 ms or if QTc prolongation is >60 ms above baseline and should be resumed at a reduced dose once QTc normalizes.^27^

Patients with CVD, especially atrial fibrillation, have a higher risk of venous thrombosis than the general population, and the presence of metastatic cancer and treatment with antiangiogenic MKI increase this risk.^22^ Therefore, in patients who are receiving prophylaxis or anticoagulant therapy, continuation is recommended dependent on comorbidities, bleeding risk, platelet count (≥50,000/mm^3^), life expectancy, and patient values and preferences.^22^ Although the drug with the highest level of evidence in cancer‐associated thrombosis is low molecular weight heparin, current trials with direct oral anticoagulants suggest that these new drugs are effective and safe in patients with cancers associated with a low risk of bleeding.^22^ Lenvatinib should be used with caution in patients who have suffered arterial thromboembolism in the previous 6 months and should be permanently discontinued after an arterial thromboembolic event.^19^, ^25^, ^27^

## 5 DISCUSSION

CV toxicity, especially hypertension, is the most frequent event in patients with RR‐DTC treated with MKI‐targeting VEGFR, and the risk is greater in those with a history of CVD, CV risk factors, or previous hypertension. Before initiating treatment, a cardiological evaluation and BP control should be carried out and a healthy lifestyle should be encouraged. Periodic monitoring of BP and cardiac function, and intensive and early treatment of any CV toxicity, are essential to maintain treatment and dose intensity until progression.

## 6 CONCLUSION

Hypertension is a common AE in patients with RR‐DCT treated with MKI‐targeting VEGF, so its prevention and intensive management are important. Before starting MKI, a healthy lifestyle, control of BP, and CV risk factors should be promoted. During treatment with MKI, if the patient develops grade 1–2 hypertension or proteinuria, an ACEi should be prescribed; if the patient is already taking an ACEi, the addition of a CCB or BAA should be considered. In case of severe toxicity (grade 3–4), the MKI should be discontinued, symptomatic treatment should be intensified, and, if the patient recovers without sequelae, the MKI should be restarted at a reduced dose and with close follow‐up.

## CONFLICT OF INTEREST

Dr Paula Jimenez‐Fonseca has received research funding, honoraria, and nonfinancial or other support from Advanced Accelerator Applications, Bristol, Eisai, HRA Pharma, Ipsen, Leo Pharma, Rovi, Sanofi, and Viatris.

## Data Availability

Data sharing is not applicable to this article as no new data were created or analyzed in this study.
